# Aspirin induces cell death and caspase-dependent phosphatidylserine externalization in HT-29 human colon adenocarcinoma cells

**DOI:** 10.1038/sj.bjc.6690690

**Published:** 1999-09

**Authors:** E Castaño, M Dalmau, M Barragán, G Pueyo, R Bartrons, J Gil

**Affiliations:** Unitat de Bioquímica, Departament de Ciències Fisiològiques II, Universitat de Barcelona, Campus de Bellvitge, L'Hospitalet, 08907, Spain; Química Farmacéutica Bayer SA, Division Consumer Care, Barcelona, Spain

**Keywords:** aspirin, apoptosis, caspases, colon cancer, HT-29

## Abstract

The induction of cell death by aspirin was analysed in HT-29 colon carcinoma cells. Aspirin induced two hallmarks of apoptosis: nuclear chromatin condensation and increase in phosphatidylserine externalization. However, aspirin did not induce either oligonucleosomal fragmentation of DNA, decrease in DNA content or nuclear fragmentation. The effect of aspirin on Annexin V binding was inhibited by the caspase inhibitor Z-VAD.fmk, indicating the involvement of caspases in the apoptotic action of aspirin. However, aspirin did not induce proteolysis of PARP, suggesting that aspirin does not increase nuclear caspase 3-like activity in HT-29 cells. This finding may be related with the ‘atypical’ features of aspirin-induced apoptosis in HT-29 cells. © 1999 Cancer Research Campaign


*British Journal of Cancer* (1999) **81**(2), 294–299
© 1999 Cancer Research Campaign
Article no. bjoc.1999.0690

### Aspirin induces cell death and caspase-dependent phosphatidylserine externalization in HT-29 human colon adenocarcinoma cells

**E Castaño[1], M Dalmau[1], M Barragán[1], G Pueyo[2], R Bartrons[1] and J Gil[1]**

[1]Unitat de Bioquímica, Departament de Ciències Fisiològiques II, Universitat de Barcelona, Campus de Bellvitge, 08907 L'Hospitalet, Spain; [2]Química Farmacéutica Bayer SA, Division Consumer Care, Barcelona, Spain

**Summary** The induction of cell death by aspirin was analysed in HT-29 colon carcinoma cells. Aspirin induced two hallmarks of apoptosis: nuclear chromatin condensation and increase in phosphatidylserine externalization. However, aspirin did not induce either oligonucleosomal fragmentation of DNA, decrease in DNA content or nuclear fragmentation. The effect of aspirin on Annexin V binding was inhibited by the caspase inhibitor Z-VAD.fmk, indicating the involvement of caspases in the apoptotic action of aspirin. However, aspirin did not induce proteolysis of PARP, suggesting that aspirin does not increase nuclear caspase 3-like activity in HT-29 cells. This finding may be related with the 'atypical' features of aspirin-induced apoptosis in HT-29 cells.

© 1999 Cancer Research Campaign

**Keywords:** aspirin; apoptosis; caspases; colon cancer; HT-29

Epidemiological studies, clinical observations and animal studies demonstrate that non-steroidal anti-inflammatory drugs (NSAIDs) can prevent colorectal cancer (reviewed in Shiff and Rigas, 1997; Smalley and DuBois, 1997). However, the mechanism for reduction of colorectal cancer by NSAIDs is not completely understood. Different mechanisms have been proposed, including inhibition of cell proliferation, inhibition of carcinogen production, potentiation of immune response, inhibition of angiogenesis and induction of apoptosis (Shiff and Rigas, 1997; Tsujii et al, 1998). These mechanisms are not exclusive and the effect of NSAIDs on the biology of cancer cells may depend on a combination of them.

Apoptosis is critical for the control of colon epithelial cell number (Hall et al, 1994). The evidence obtained in recent years indicates that many cancer chemotherapy agents induce apoptosis of tumour cells (Hannun, 1997). NSAIDs induce apoptosis of different cell types, including human colorectal tumour cell lines (Piazza et al, 1995; Shiff et al, 1995; Elder et al, 1996) and rat enterocytes (Arber et al, 1997). Furthermore, administration of NSAIDs induces apoptosis of colon cancer cells in vivo (Pasricha et al, 1995; Boolbol et al, 1996). The mechanism responsible for this apoptotic effect of NSAIDs is not clear. Aspirin and other NSAIDs directly target cyclooxygenase (COX) (Vane, 1971), a key enzyme in the production of prostaglandins, prostacyclins and thomboxanes (Smith, 1989). Although COX is the molecular target of most NSAIDs, both COX-dependent and COX-independent mechanisms in the apoptotic action of NSAIDs have been reported (Shiff and Rigas, 1997; Elder and Paraskeva, 1998; Gupta and DuBois, 1998).

Whether or not aspirin induces apoptosis is still controversial. It has been reported that aspirin, in contrast to other NSAIDs, does not induce apoptosis either in colon carcinoma HT-29 cells (Shiff et al, 1996; Piazza et al, 1997), or in v-src-transformed chicken fibroblasts (Lu et al, 1995). However, we found that in B-CLL cells aspirin induces all the typical features of apoptosis, including internucleosomal DNA fragmentation, decrease in DNA content, increase in phosphatidylserine exposure and proteolysis of poly(ADPribose) polymerase (PARP) (Bellosillo et al, 1998). Consistent with our results, it has been reported that aspirin induces apoptosis of colon adenocarcinoma Caco-2 cells (Ricchi et al, 1997), HT-29 cells (Qiao et al, 1998) and crypt cells (Barnes et al, 1998). The apoptotic action of aspirin on B-CLL cells was inhibited by the caspase inhibitor Z-VAD.fmk, demonstrating the involvement of caspases (Bellosillo et al, 1998). Caspases are responsible for many of the biochemical and morphological processes during apoptosis, including DNA fragmentation, phosphatidylserine exposure, degradation of nuclear lamins, nuclear morphological changes, and proteolysis of PARP and other enzymes involved in DNA repair and genomic stability (Cohen, 1997; Cryns and Yuan, 1998).

In HT-29 cells, the cell death induced by aspirin was associated neither with a ladder pattern in genomic DNA electrophoresis nor with a subdiploid peak in flow cytometry, so it was considered 'atypical apoptosis' (Qiao et al, 1998). A very recent report concludes that necrosis rather than apoptosis is the mechanism that accounts for aspirin toxicity in human colon SW 620 and HT-29 cells (Subbegowda and Frommel et al, 1998).

Aspirin is one of the most widely studied NSAIDs in the prevention of colon cancer. Thus, from the viewpoint of colon cancer chemotherapy it is important to reveal the mechanism of aspirin-induced cell death. Furthermore, for in vitro studies of colon cancer chemotherapy-induced apoptosis, the human colonic adenocarcinoma cell line HT-29 is a valuable tool. The aim of this study was to further analyse the induction of cell death by aspirin in HT-29 cells, focusing on the effect of aspirin on the activation of caspases.

*Received 13 August 1998*
*Revised 22 March 1999*
*Accepted 31 March 1999*

*Correspondence to:* J Gil

### MATERIALS AND METHODS

### Reagents

Aspirin (acetylsalicylic acid), 3,(4,5-dimethylthiazol-2-yl)2,4-diphenyltetrazolium bromide (MTT) and propidium iodide were obtained from Sigma (St Louis, MO, USA). Fetal calf serum (FCS) was from Gibco Laboratories and Dulbecco's modified Eagle's medium (DMEM) was from Biological Industries. *N*-benzyloxycarbonyl-Val-Ala-Asp-fluoromethyl ketone (Z-VAD.fmk) was obtained from Enzyme Systems Products (Dublin, CA, USA). Annexin V-FITC was obtained from Boehinger Ingelheim. PARP polyclonal antibody (Vi.5) raised against the recombinant human PARP over-produced in Sf 9/baculovirus was kindly provided by Dr Gilbert de Murcia (Strasbourg, France). All the other reagents were of analytical grade.

### Cell culture

The human colon adenocarcinoma cell line HT-29 (ATCC HTB 28) was obtained from the European Type Culture Collection. Stock cultures of cells were maintained in DMEM supplemented with 10% FCS, L-glutamine (2 mM), penicillin (100 units $ml^{-1}$) and streptomycin (100 μg $ml^{-1}$) in a humidified atmosphere of 10% carbon dioxide/90% air at 37°C. To obtain cultures for experimental purposes, cells were plated at 25 000 or 50 000 cells $cm^{-2}$. Assays using preconfluent conditions were treated by adding the appropriate amount of drug stock solution directly to the media in absence of FCS the day after plating at the described densities.

### Cell number assay

Cell number was determined by the MTT assay (Mosmann, 1983). HT-29 cells (20 000 cells per well) were incubated in 24-well plates in the absence or in the presence of factors in a final volume of 500 μl. At different times, 50 μl of MTT (5 mg $ml^{-1}$ in phosphate-buffered saline (PBS) was added to each well for a further 3 h. The blue MTT formazan precipitated was dissolved in 500 μl of isopropanol: 1 M hydrochloric acid (24:1) and the absorbance values at 570 nm were determined on a multiwell plate reader.

### Apoptosis

In all experiments floating and freshly trypsinized cells were pooled and apoptosis was evaluated by the following assays.

#### *Measurement of the annexin-V and propidium iodide staining by FACS analysis*

Cells were washed twice in binding buffer (10 mM HEPES–sodium hydroxide pH 7.4, 140 mM sodium chloride, 2.5 mM calcium chloride, and resuspended in the same buffer at $10^6$ cells $ml^{-1}$ in the presence of 0.5 μl of annexin-V-FITC. After 30 min of incubation at room temperature, propidium iodine (PI) was added at 0.05 μg $ml^{-1}$. The fluorescence of cells was analysed by flow cytometry (FACSCalibur, Becton Dickinson, Mountain View, CA, USA), using the Cell Quest software.

#### *Measurement of cell detachment*

HT-29 cells (200 000 cells per well) were incubated in 35-mm dishes in the absence or in the presence of factors in a final volume of 2 ml. The total number of floating and attached cells was determined by microscopy using the Neubauer micro slide.

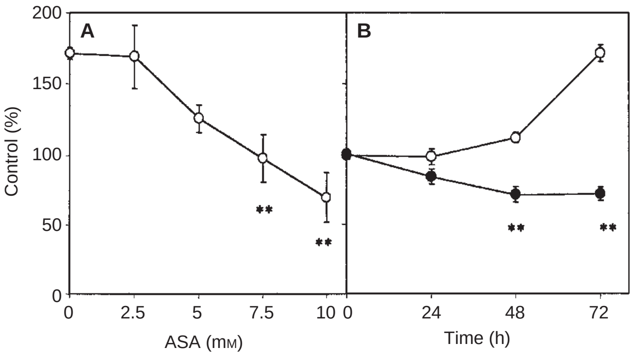

**Figure 1** Cytotoxic effect of aspirin on HT-29 cells. (**A**) Dose–response of the cytotoxic effect of aspirin on HT-29 cells. Cells were incubated for 72 h with various concentrations of aspirin, ranging from 0 to 10 mM. Statistical significance of differences was assessed between different concentrations of aspirin vs control. Data are shown as the mean value ± SEM of five independent experiments performed in triplicate. (**B**) Time-course of aspirin-induced cytotoxicity on HT-29 cells. Cells were incubated without (○) or with 10 mM aspirin (●) (ASA) for the times indicated. Statistical significance of differences was assessed with respect to time zero. Cell viability was determined by the MTT assay as described in Materials and Methods. The results are expressed as a percentage of the optical density produced by the starting number of cells. Data are shown as the mean value ± s.e.m. of six independent experiments

### Western blot analysis of PARP

Cells were plated onto 100 $cm^2$ dishes at a density of $5 \times 10^6$ cells per dish. After washing the cultures with PBS, they were lysed with Laemmli sample buffer (Laemmli, 1970) and samples were incubated for 10 min at 100°C.

Protein concentration was determinated by the BCA Protein Assay (Pierce, Rockford, IL, USA). Fifty micrograms of the protein extract was subjected to sodium dodecyl sulphate polyacrylamide gel electrophoresis (SDS-PAGE), and transferred to Immobilon-P membranes (Millipore, Bedford, MA, USA). After blocking for 1 h with 5% dried skimmed milk in TBST (50 mM Tris–HCl pH 8, 150 mM sodium chloride, 0.5% Tween-20), the filters were incubated with Vi.2 antibody diluted 1:1000 in 5% dried skimmed milk in TBST. Antibody binding was detected by using a secondary antibody (anti-rabbit immunoglobulin, Amersham, Buckinghamshire, UK) conjugated to horseradish peroxidase diluted 1:5000 in 5% dried skimmed milk in TBST and an enhanced chemiluminescence (ECL) detection kit (Amersham, Buckinghamshire, UK).

### Data analysis

All data points shown are mean values ± s.e.m. of *n* independent experiments. The data points from duplicates or triplicates of an individual experiments were averaged, and the data points shown are the mean of these averages from *n* experiments. Statistical significance of differences was assessed by ANOVA (Fisher PLSD test). Differences between absence and presence of aspirin are indicated by (*) $P < 0.05$, (**) $P < 0.01$ and (***) $P < 0.001$.

### RESULTS

### Cytotoxic effect of aspirin on HT-29 cells

We first studied the cytotoxic effect of aspirin on HT-29 cells. Cells were incubated for 72 h with different aspirin concentrations,

*© 1999 Cancer Research Campaign*

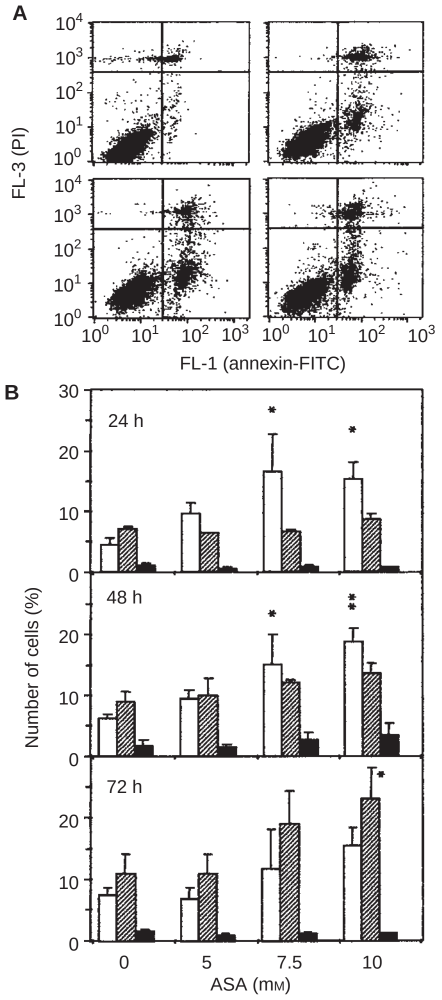

**Figure 2** Analysis of apoptosis by the annexin V binding assay. HT-29 cells were treated with different concentrations of aspirin, which ranged from 0 to 10 mM. Annexin V binding was quantified as described in Materials and Methods. (**A**) Representative dot plots of annexin V versus PI fluorescence for control cells (a), and cells treated with 5 (b), 7.5 (c) and 10 mM (d) ASA for 24 h. (**B**) Percentage of cells annexin V + / PI – (open bars), annexin V + / PI + (dashed bars) and annexin V – / PI + (closed bars) after 24, 48 and 72 h of treatment. Data shown are the mean values ± s.e.m. of two independent experiments performed in duplicate

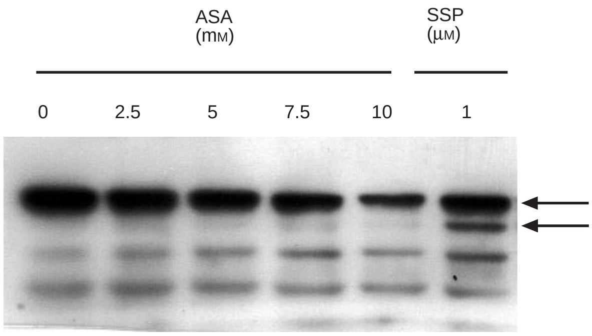

**Figure 3** Effect of aspirin on PARP cleavage. Cells were incubated for 72 h with 0–10 mM aspirin (ASA) or 1 μM of staurosporin. PARP cleavage was analysed on protein extracts from these cells by Western blot as described in Materials and Methods. The position of native PARP (116 kDa) and the proteolytic fragment (85 kDa) is indicated ranging from 0 to 10 mM, and the number of viable cells was determined by the MTT assay. The decrease in cell number was dose-dependent (Figure 1A). The mean $IC_{50}$ of these dose–response studies was 5 mM. In agreement with previous studies (Shiff et al, 1996; Qiao et al, 1998), aspirin treatment induced an increase in the G0/G1 phase of the cell cycle (results not shown). As aspirin inhibits proliferation, only a decrease in the number of cells with respect to time zero necessarily implies an increase in cell death. Thus, the cytotoxicity of aspirin on HT-29 cells was studied with time-course assays. A significant increase in cell death was observed after 48 h of incubation with 10 mM aspirin (Figure 1B).

### Characterization of aspirin-induced cell death in HT-29 cells

We next studied the characteristics of aspirin-induced cell death in HT-29 cells. The appearance of apoptotic cells was analysed in treated cultures according to morphological criteria (Duke and Cohen, 1992). The addition of aspirin to HT-29 cells induced morphological changes characteristic of apoptosis, including cell shrinkage and strong condensation of nuclei (data not shown). However, nuclear fragmentation or apoptotic bodies were not observed. Consistent with a previous report (Qiao et al, 1998), cells treated with several concentrations of aspirin did not show evidence of a ladder pattern of their genomic DNA when analysed by agarose gel electrophoresis, nor a decrease in DNA content when analysed by flow cytometry (data not shown).

To corroborate the induction of apoptosis by aspirin in HT-29 cells, the externalization of phosphatidylserine was studied with the annexin V-binding assay (van Engeland et al, 1998). The affinity of annexin V for the PS residues allows the percentage of cells undergoing apoptosis to be quantified by flow cytometry. Apoptotic and necrotic cells were distinguished on the basis of a double-labelling for annexin V-FITC and PI, a membrane impermeable DNA stain. Aspirin induced a dose-dependent increase in the percentage of apoptotic cells (annexin V-positive/PI-negative), when treated for 24 h (Figure 2). In contrast, the number of necrotic (annexin V-positive/PI-positive) and damaged (annexin V-negative/PI-positive) cells was not significantly increased ($P > 0.05$). At 48 h and 72 h an increase in the double positive population was detected (Figure 2B). This effect is consistent with the secondary necrosis process, which usually comes after apoptosis in cell cultures. These results demonstrate that aspirin induces apoptosis of HT-29 cells.

### Involvement of caspases in the apoptotic effect of aspirin

We analysed whether incubation of HT-29 cells with aspirin induced proteolytic cleavage of PARP, a hallmark of activation of caspase-3 like proteases during apoptosis. Incubation of HT-29 cells for 72 h with various doses of aspirin, ranging from 2.5 to 10 mM, induced a decrease in the cellular levels of PARP without detectable proteolysis (Figure 3). Proteolytic cleavage of PARP was not detected at 24 h or at 48 h (data not shown). As positive controls we used HT-29 cells treated with 1 μM staurosporine (Figure 3) and aspirin-treated B-CLL cells (data not shown) which revealed the 85 kDa fragment of PARP.

Apoptosis-induced PS exposure is prevented by caspase inhibitors (Martin et al, 1996; Vanags et al, 1996). In order to

*© 1999 Cancer Research Campaign*

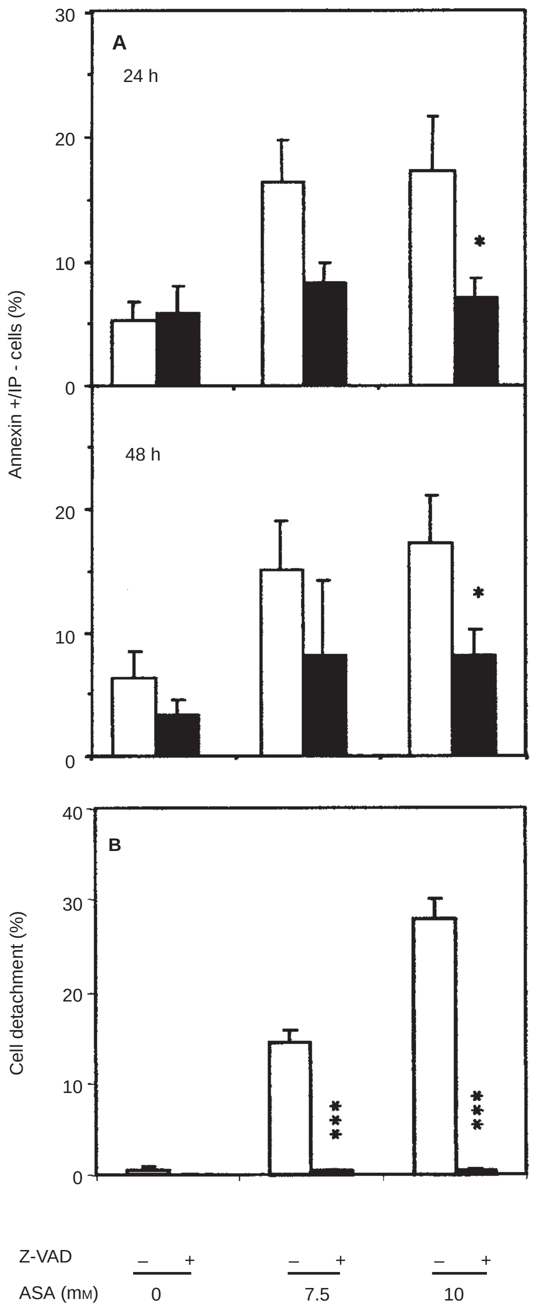

**Figure 4** Effect of the caspase inhibitor Z-VAD.fmk on aspirin-induced apoptosis in HT-29 cells. (**A**) Effect of the caspase inhibitor Z-VAD.fmk on the annexin-V binding of HT-29 cells. Cells were incubated for 24 or 48 h either alone or in the presence of aspirin (ASA) (0, 7.5 or 10 mM) (open bars) or aspirin with 200 μM Z-VAD.fmk (solid bars). Z-VAD.fmk was added 1 h prior to aspirin administration. Annexin-V binding was quantified as described in Materials and Methods. (**B**) Effect of the caspase inhibitor Z-VAD.fmk on cell detachment. Cells were incubated for 48 h either alone or in the presence of aspirin (ASA) (0, 7.5 or 10 mM) (open bars) or aspirin with 200 μM Z-VAD.fmk (solid bars). Z-VAD.fmk was added 1 h before aspirin administration. The data indicate the quantity of floating cells as a proportion of the total cell number (attached and floating). Statistical significance of differences was assessed between absence and presence of Z-VAD.fmk. Data are shown as the mean value ± s.e.m. of three independent experiments made in triplicate assess the involvement of caspases in the apoptotic effect of aspirin, we studied whether the caspase inhibitor Z-VAD.fmk prevented aspirin-induced PS externalization. HT-29 cultures were incubated for 24 h and 48 h with different concentrations of aspirin in the presence or absence of 200 μM Z-VAD.fmk, which had no cytotoxic effects. Z-VAD.fmk prevented the increase in annexin+/IP−cells induced by aspirin at all concentrations used (Figure 4A). Furthermore, Z-VAD.fmk also prevented aspirin-induced cell detachment in HT-29 cells (Figure 4B).

### DISCUSSION

The results shown in this report demonstrate that aspirin induces caspase-dependent externalization of phosphatidylserine in HT-29 cells. It has been described that the loss of membrane asymmetry during the apoptotic process results in the exposure of phosphatidylserine (PS) at the outer plasma membrane leaflet of the cell, a fundamental characteristic that distinguishes apoptosis from necrosis (reviewed in Van Engeland et al, 1998). This is a very early phenomenon, which follows caspase activation but probably precedes nuclear condensation. Aspirin-induced PS externalization can be blocked by caspase inhibitors, demonstrating the involvement of caspases. Furthermore, in agreement with previous reports (Elder et al, 1996; Qiao et al, 1998), aspirin induced cell detachment and morphological changes characteristic of apoptosis in HT-29 cells. Considering these results, we can conclude that aspirin induces an apoptotic process in HT-29 colonic carcinoma cells. Although HT-29 cells express caspase-3, we did not detect cleavage of PARP by caspase-3-like proteases during aspirin-induced apoptosis in HT-29 cells. This finding could be related with the ‘atypical’ apoptotic features of aspirin-induced cell death in these cells. Some cell types from caspase-3 knockout mice are incapable of DNA degradation, but display other hallmarks of apoptosis, like PS externalization (Woo et al, 1998). Furthermore, the MCF-7 breast carcinoma cell line, which does not express caspase-3 (Janicke et al, 1998; Zapata et al, 1998), undergoes apoptosis in the absence of DNA fragmentation (Oberhammer et al, 1993; Janicke et al, 1998), but showing PS externalization (Mangiarotti et al, 1998). Interestingly, MCF-7 cells treated with the NSAID sulindac show nuclear condensation without clear DNA ladder (Han et al, 1998).

The inability of aspirin to induce some of the biochemical characteristics of apoptosis in HT-29 cells may be signal-specific, since 7-hydroxystaurosporine (Shao et al, 1997), butyrate (Heerdt et al, 1994), Fas (Bonnotte et al, 1998) and intestinal trefoil factor 3 (Efstathiou et al, 1998) induce oligonucleosomal fragmentation of DNA in HT-29 cells. On the other hand, nitrogen mustard induces apoptosis of HT-29 cells in the absence of DNA laddering (Boddie et al, 1998).

The mechanisms by which NSAIDs induce apoptosis are not clear. Consistent with the hypothesis that the apoptotic action of NSAIDs is mediated by the inhibition of COX, overexpression of COX-2 in rat epithelial intestinal cells inhibits butyrate-induced apoptosis and this inhibition was reversed by the NSAID sulindac sulphide (Tsujii and DuBois, 1995). However, the sulfone metabolite of sulindac, which does not inhibit cyclooxygenases, also induces apoptosis of HT-29 cells (Piazza et al, 1995), and COX-2 selective inhibitors induce apoptosis in human colorectal carcinoma cell lines independently of COX-2 protein expression (Elder et al, 1997). Taken together these results suggest the contribution of COX-dependent and COX-independent mechanisms in the apoptotic action of NSAIDs. Interestingly, we have recently demonstrated that aspirin inhibits DNA synthesis in Swiss 3T3 fibroblasts by both COX-dependent and COX-independent mechanisms, depending on the concentration used (Castaño et al, 1997).

*© 1999 Cancer Research Campaign* 

Cyclooxygenase-independent mechanisms have been implicated in the inhibition of the transcription factor NF-κB by aspirin and salicylate (Kopp and Ghosh, 1994; Schwenger et al, 1998). Very recently, it has been reported that aspirin binds and inhibits IκB kinase β (Yin et al, 1998). Remarkably, NF-κB has an essential role in preventing tumour necrosis factor α and cancer therapy-induced apoptosis (Liu et al, 1996; Van Antwerp et al, 1996; Wang et al, 1996; Beg and Baltimore, 1996). The elucidation of the mechanisms involved in the apoptotic action of aspirin in HT-29 cells needs further investigation.

Many cancer cells demonstrate reduced capabilities of responding to apoptotic stimuli (Hoffman and Liebermann, 1994). The unusual features of aspirin-induced cell death in HT-29 cells may offer clues to the mechanisms by which cancer cells escape from apoptosis.

### ACKNOWLEDGEMENTS

The authors thank M Vallés for technical assistance, M Piqué, Dr F Ventura, B Bellosillo and Dr G Pons for comments, helpful discussions and suggestions. We also thank Dr Gilbert de Murcia for kindly providing anti PARP polyclonal antibody, Dr X Graña, Dr I Fabregat and Dr JM Zapata for critical reading, Dr J Canela for statistical assistance and R Rycroft for language assistance. EC is supported by a Fellowship from Universitat de Barcelona, MB was supported by a Fellowship from Fundació August Pi i Sunyer. This work was supported by grants from Química Farmacéutica Bayer SA (Division Consumer Care), 'Marató de TV3' the 'Fundació August Pi i Sunyer' (Campus de Bellvitge) and 'Generalitat de Catalunya' (97SRG/74).

### REFERENCES

Arber N, Han EK, Sgambato A, Piazza GA, Delohery TM, Begemann M, Weghorst CM, Kim NH, Pamukcu R, Ahnen DJ, Reed JC, Weinstein IB and Holt PR (1997) A K-ras oncogene increase resistance to sulindac-induced apoptosis in rat enterocytes. *Gastroenterology* **113**: 1892–1900

Barnes CJ, Cameron IL, Hardman WE and Lee M (1998) Non-steroidal anti-inflammatory drug effect on crypt cell proliferation and apoptosis during initiation of rat colon carcinogenesis. *Br J Cancer* **77**: 573–580

Bellosillo B, Pique M, Barragan M, Castaño E, Villamor N, Colomer D, Montserrat E, Pons G and Gil J (1998). Aspirin and salicylate induce apoptosis and activation of caspases in B-cell chronic lymphocytic leukemia cells. *Blood* **92**: 1406–1414

Beg AA and Baltimore D (1996) An essential role for NF-κB in preventing TNF-α-induced cell death. *Science* **274**: 782–784

Boddie AW, Constantinou A, Williams C and Reed A (1998) Nitrogen mustard up-regulates Bcl-2 and GSH and increases NTP and PCr in HT-29 colon cancer cells. *Br J Cancer* **77**: 1395–1404

Bonnotte B, Favre N, Reveneau S, Micheau O, Droin N, Garrido C, Fontana A, Chauffert B, Solary E and Martin F (1998) Cancer cell sensitization to Fas-mediated apoptosis by sodium butyrate. *Cell Death and Differ* **5**: 480–487

Boolbol SK, Dannenberg AJ, Chadburn A, Martucci C, Guo XJ, Ramonetti JT, Abreu-Goris M, Newmark HL, Lipkin ML, DeCosse JJ and Bertagnolli MM (1996) Cyclooxygenase-2 overexpression and tumor formation are blocked by sulindac in a murine model of familial adenomatous polyposis. *Cancer Res* **56**: 2556–2560

Castaño E, Dalmau M, Marti M, Berrocal F, Bartrons R and Gil J (1997) Inhibition of DNA synthesis by aspirin in Swiss 3T3 cell fibroblasts. *J Pharmacol Exp Ther* **280**: 366–372

Cohen GM (1997) Caspases: the executioners of apoptosis. *Biochem J* **326**: 1–16

Cryns V and Yuan J (1998) Proteases to die for. *Genes Dev* **12**: 1551–1557

Duke RC and Cohen JJ (1992) Morphological and biochemical assays of apoptosis. In: *Current Protocols in Immunology*, Coligan JE and Kruisbeak AM (eds), pp. 3.17.1–3.17.16. John Wiley & Sons: New York

Efstathiou JA, Noda M, Rowan A, Dixon C, Chinery R, Jawhari A, Hattori T, Wright NA, Bodmer WF and Pignatelli M (1998) Intestinal trefoil factor controls the expression of the adenomatous polyposis coli-catenin and E-cadherin-catenin complexes in human colon carcinoma cells. *Proc Natl Acad Sci USA* **95**: 3122–3127

Elder DJ and Paraskeva C (1998) COX-2 inhibitors for colorectal cancer. *Nat Med* **4**: 392–393

Elder DJ, Hague A, Hicks DJ and Paraskeva C (1996) Differential growth inhibition by the aspirin metabolite salicylate in human colorectal tumor cell lines: enhanced apoptosis in carcinoma and in vitro-transformed adenoma relative to adenoma cell lines. *Cancer Res* **56**: 2273–2276

Elder DJE, Halton DE, Hague A and Paraskeva C (1997) Induction of apoptotic cell death in human colorectal carcinoma cell lines by a cyclooxygenase 2 (COX-2) selective nonsteroidal anti inflammatory drug: independence from COX 2 protein expression. *Clin Cancer Res* **3**: 1679–1983

Gupta RA and DuBois RN (1998) Aspirin, NSAIDs, and colon carcer prevention: mechanisms? *Gastroenterology* **114**: 1095–1100

Hall PA, Coates PJ, Ansari B and Hopwood D (1994) Regulation of cell number in the mammalian gastrointestinal tract: the importance of apoptosis. *J Cell Sci* **107**: 3569–3577

Heerdt BG, Houston MA and Augenlicht LH (1994) Potentiation by specific short-chain fatty acids of differentiation and apoptosis in human colonic carcinoma cell lines. *Cancer Res* **54**: 3288–3294

Han EK, Arber N, Yamamoto H, Lim JT, Delohery T, Pamukcu R, Piazza GA, Xing WQ and Weinstein IB (1998) Effects of sulindac and its metabolites on growth and apoptosis in human mammary epithelial and breast carcinoma cell lines. *Breast Cancer Res Treat* **48**: 195–203

Hannun YA (1997) Apoptosis and the dilemma of cancer chemotherapy. *Blood* **89**: 1845–1853

Hoffman B and Liebermann DA (1994) Molecular control of apoptosis: differentiation/growth arrest primary response genes, proto-oncogenes, and tumor supressor genes as positive and negative modulators. *Oncogene* **9**: 1807–1812

Janicke RU, Sprengart ML, Wati MR and Porter AG (1998) Caspase-3 is required for DNA fragmentation and morphological changes associated with apoptosis. *J Biol Chem* **273**: 9357–9360

Kopp E and Ghosh S (1994) Inhibition of NF-κB by sodium salicylate and aspirin. *Science* **265**: 956–959

Laemmli UK (1970) Cleavage of structural proteins during the assembly of the head of bacteriophage T4. *Nature* **227**: 680–685

Liu ZG, Hsu H, Goeddel DV and Karin M (1996) Dissection of TNF receptor 1 effector functions. JNK activation is not linked to apoptosis while NF-κB activation prevents cell death. *Cell* **87**: 565–576

Lu X, Xie W, Reed D, Bradshaw WS and Simmons DL (1995) Nonsteroidal antiinflammatory drugs cause apoptosis and induce cyclooxygenases in chicken embryo fibroblasts. *Proc Natl Acad Sci USA* **92**: 7961–7965

Mangiarotti R, Danova M, Alberici R and Pellicciari C (1998) All-trans retinoic acid (ATRA)-induced apoptosis is preceded by G1 arrest in human MCF-7 breast cancer cells. *Br J Cancer* **77**: 186–191

Martin SJ, Finucane DM, Amarante-Mendes GP, O'Brien GA and Green DR (1996) Phosphatidylserine externalization during CD95-induced apoptosis of cells and cytoplasts requires ICE/CED-3 protease activity. *J Biol Chem* **271**: 28753–28756

Mosmann T (1983) Rapid colorimetric assay for cellular growth and survival: application to proliferation and cytotoxicity assays. *J Immunol Methods* **65**: 55–63

Oberhammer F, Wilson JW, Dive C, Morris ID, Hickman JA, Wakeling AE, Walker PR and Sikorska M (1993) Apoptotic death in epithelial cells: cleavage of DNA to 300 and/or 50 kb fragments prior to or in the absence of internucleosomal fragmentation. *EMBO J* **12**: 3679–3684

Pasricha PJ, Bedi A, O'Connor K, Rashid A, Akhtar AJ, Zahurak ML, Piantadosi S, Hamilton SR and Giardiello FM (1995) The effects of sulindac on colorectal proliferation and apoptosis in familial ademonatous polyposis. *Gastroenterology* **109**: 994–998

Piazza GA, Rahm AL, Krutzsch M, Sperl G, Paranka NS, Gross PH, Brendel K, Burt RW, Alberts DS, Pamukcu R and Ahnen DJ (1995) Antineoplastic drugs sulindac sulfide and sulfone inhibit cell growth by inducing apoptosis. *Cancer Res* **55**: 3110–3116

Piazza GA, Rahm AK, Finn TS, Fryer BH, Li H, Stoumen AL, Pamukcu R and Ahnen DJ (1997) Apoptosis primarily accounts for the growth-inhibitory properties of sulindac metabolites and involves a mechanism that is independent of cyclooxygenase inhibition, cell cycle arrest, and p53 induction. *Cancer Res* **57**: 2452–2459

 © 1999 Cancer Research Campaign

Qiao L, Hanif R, Sphicas E, Shiff SJ and Rigas B (1998) Effect of aspirin on induction of apoptosis in HT-29 human colon adenocarcinoma cells. *Biochem Pharmacol* **55**: 53–64

Ricchi P, Pignata S, Dipopolo A, Memoli A, Apicella A, Zarrilli R and Acquaviva AM (1997) Effect of aspirin on cell proliferation and differentiation of colon adenocarcinoma Caco-2 cells. *Int J Cancer* **73**: 880–884

Schwenger P, Alpert D, Skolnik EY and Vilcek J (1998) Activation of p38 mitogen-activated protein kinase by sodium salicylate leads to inhibition of tumor necrosis factor-induced I kappaB alpha phosphorylation and degradation. *Mol Cell Biol* **18**: 78–84

Shao RG, Shimizu T and Pommier Y (1997) 7-Hydroxystaurosporine (UCN-01) induces apoptosis in human colon carcinoma and leukemia cells independently of p53. *Exp Cell Res* **234**: 388–397

Shiff SJ and Rigas B (1997) Nonsteroidal anti-inflammatory drugs and colorectal cancer: evolving concepts of their chemopreventive actions. *Gastroenterology* **113**: 1992–1998

Shiff SJ, Qiao L, Tsai LL and Rigas B (1995) Sulindac sulfide, an aspirin-like compound, inhibits proliferation, causes cell cycle quiescence, and induces apoptosis in HT-29 colon adenocarcinoma cells. *J Clin Invest* **96**: 491–503

Shiff SJ, Koutsos MI, Qiao L and Rigas B (1996) Non steroidal antiinflammatory drugs inhibit the proliferation of colon adenocarcioma cells: effects on cell cycle and apoptosis. *Exp Cell Res* **222**: 179–188

Smalley WE and DuBois RN (1997) Colorectal cancer and nonsteroidal anti-inflammatory drugs. *Adv Pharmacol* **39**: 1–20

Smith WL (1989) The eicosanoids and their biochemical mechanism of action. *Biochem J* **259**: 315–324

Subbegowda R and Frommel TO (1998) Aspirin toxicity for human colonic tumor cells results from necrosis and is accompanied by cell cycle arrest. *Cancer Res* **58**: 2772–2776.

Tsujii M and Dubois RN (1995) Alterations in cellular adhesion and apoptosis in epithelial cells overexpressing prostaglandin endoperoxide synthase 2. *Cell* **83**: 493–501

Tsujii M, Kawano S, Tsuji S, Sawaoka H, Hori M and DuBois RN (1998) Cyclooxygenase regulates angiogenesis induced by colon cancer cells. *Cell* **93**: 705–716

Vanags DM, Porn-Ares MI, Coppola S, Burgess DH and Orrenius S (1996) Protease involvement in fodrin cleavage and phosphatidylserine exposure in apoptosis. *J Biol Chem* **271**: 31075–31085

Van Antwerp DJ, Martin SJ, Kafri T, Green DR and Verma IM (1996) Suppression of TNF-α-induced apoptosis by NF-κB. *Science* **274**: 787–789.

Van Engeland M, Nieland LJ, Ramaekers FC, Schutte B and Reutelingsperger CP (1998) Annexin V-affinity assay: a review on an apoptosis detection system based on phosphatidylserine exposure. *Cytometry* **31**: 1–9

Vane JR (1971) Inhibition of prostaglandin synthesis as a mechanism of action for aspirin-like drugs. *Nature New Biol* **231**: 232–235

Wang CY, Mayo MW and Baldwin AS (1996) TNF and cancer therapy-induced apoptosis: potentiation by inhibition of NF-κB in preventing TNF-α-induced cell death. *Science* **274**: 784–787

Woo M, Hakem R, Soengas MS, Duncan GS, Shahinian A, Kagi D, Hakem A, McCurrach M, Khoo W, Kaufman SA, Senaldi G, Howard T, Lowe SW and Mak TW (1998) Essential contribution of caspase 3/CPP32 to apoptosis and its associated nuclear changes. *Genes Dev* **12**: 806–819

Yin MJ, Yamamoto Y and Gaynor RB (1998) The anti-inflammatory agents aspirin and salicylate inhibit the activity of I(kappa)B kinase-beta. *Nature* **396**: 77–80

Zapata JM, Krajewska M, Krajewski S, Huang RP, Takayama S, Wang HG, Adamson E and Reed JC (1998) Expression of multiple apoptosis-regulatory genes in human breast cancer cell lines and primary tumors. *Breast Cancer Res Treat* **47**: 129–140

*© 1999 Cancer Research Campaign*

## References

[PDF_00457] Arber N., Han E. K., Sgambato A., Piazza G. A., Delohery T. M., Begemann M., Weghorst C. M., Kim N. H., Pamukcu R., Ahnen D. J. (1997). A K-ras oncogene increases resistance to sulindac-induced apoptosis in rat enterocytes.. Gastroenterology.

[PDF_00461] Barnes C. J., Cameron I. L., Hardman W. E., Lee M. (1998). Non-steroidol anti-inflammatory drug effect on crypt cell proliferation and apoptosis during initiation of rat colon carcinogenesis.. Br J Cancer.

[PDF_00468] Beg A. A., Baltimore D. (1996). An essential role for NF-kappaB in preventing TNF-alpha-induced cell death.. Science.

[PDF_00464] Bellosillo B., Piqué M., Barragán M., Castaño E., Villamor N., Colomer D., Montserrat E., Pons G., Gil J. (1998). Aspirin and salicylate induce apoptosis and activation of caspases in B-cell chronic lymphocytic leukemia cells.. Blood.

[PDF_00470] Boddie A. W., Constantinou A., Williams C., Reed A. (1998). Nitrogen mustard up-regulates Bcl-2 and GSH and increases NTP and PCr in HT-29 colon cancer cells.. Br J Cancer.

[PDF_00473] Bonnotte B., Favre N., Reveneau S., Micheau O., Droin N., Garrido C., Fontana A., Chauffert B., Solary E., Martin F. (1998). Cancer cell sensitization to fas-mediated apoptosis by sodium butyrate.. Cell Death Differ.

[PDF_00476] Boolbol S. K., Dannenberg A. J., Chadburn A., Martucci C., Guo X. J., Ramonetti J. T., Abreu-Goris M., Newmark H. L., Lipkin M. L., DeCosse J. J. (1996). Cyclooxygenase-2 overexpression and tumor formation are blocked by sulindac in a murine model of familial adenomatous polyposis.. Cancer Res.

[PDF_00481] Castaño E., Dalmau M., Martí M., Berrocal F., Bartrons R., Gil J. (1997). Inhibition of DNA synthesis by aspirin in Swiss 3T3 fibroblasts.. J Pharmacol Exp Ther.

[PDF_00484] Cohen G. M. (1997). Caspases: the executioners of apoptosis.. Biochem J.

[PDF_00485] Cryns V., Yuan J. (1998). Proteases to die for.. Genes Dev.

[PDF_00489] Efstathiou J. A., Noda M., Rowan A., Dixon C., Chinery R., Jawhari A., Hattori T., Wright N. A., Bodmer W. F., Pignatelli M. (1998). Intestinal trefoil factor controls the expression of the adenomatous polyposis coli-catenin and the E-cadherin-catenin complexes in human colon carcinoma cells.. Proc Natl Acad Sci U S A.

[PDF_00496] Elder D. J., Hague A., Hicks D. J., Paraskeva C. (1996). Differential growth inhibition by the aspirin metabolite salicylate in human colorectal tumor cell lines: enhanced apoptosis in carcinoma and in vitro-transformed adenoma relative to adenoma relative to adenoma cell lines.. Cancer Res.

[PDF_00500] Elder D. J., Halton D. E., Hague A., Paraskeva C. (1997). Induction of apoptotic cell death in human colorectal carcinoma cell lines by a cyclooxygenase-2 (COX-2)-selective nonsteroidal anti-inflammatory drug: independence from COX-2 protein expression.. Clin Cancer Res.

[PDF_00494] Elder D. J., Paraskeva C. (1998). COX-2 inhibitors for colorectal cancer.. Nat Med.

[PDF_00505] Gupta R. A., DuBois R. N. (1998). Aspirin, NSAIDS, and colon cancer prevention: mechanisms?. Gastroenterology.

[PDF_00507] Hall P. A., Coates P. J., Ansari B., Hopwood D. (1994). Regulation of cell number in the mammalian gastrointestinal tract: the importance of apoptosis.. J Cell Sci.

[PDF_00513] Han E. K., Arber N., Yamamoto H., Lim J. T., Delohery T., Pamukcu R., Piazza G. A., Xing W. Q., Weinstein I. B. (1998). Effects of sulindac and its metabolites on growth and apoptosis in human mammary epithelial and breast carcinoma cell lines.. Breast Cancer Res Treat.

[PDF_00517] Hannun Y. A. (1997). Apoptosis and the dilemma of cancer chemotherapy.. Blood.

[PDF_00510] Heerdt B. G., Houston M. A., Augenlicht L. H. (1994). Potentiation by specific short-chain fatty acids of differentiation and apoptosis in human colonic carcinoma cell lines.. Cancer Res.

[PDF_00519] Hoffman B., Liebermann D. A. (1994). Molecular controls of apoptosis: differentiation/growth arrest primary response genes, proto-oncogenes, and tumor suppressor genes as positive & negative modulators.. Oncogene.

[PDF_00523] Jänicke R. U., Sprengart M. L., Wati M. R., Porter A. G. (1998). Caspase-3 is required for DNA fragmentation and morphological changes associated with apoptosis.. J Biol Chem.

[PDF_00526] Kopp E., Ghosh S. (1994). Inhibition of NF-kappa B by sodium salicylate and aspirin.. Science.

[PDF_00528] Laemmli U. K. (1970). Cleavage of structural proteins during the assembly of the head of bacteriophage T4.. Nature.

[PDF_00530] Liu Z. G., Hsu H., Goeddel D. V., Karin M. (1996). Dissection of TNF receptor 1 effector functions: JNK activation is not linked to apoptosis while NF-kappaB activation prevents cell death.. Cell.

[PDF_00533] Lu X., Xie W., Reed D., Bradshaw W. S., Simmons D. L. (1995). Nonsteroidal antiinflammatory drugs cause apoptosis and induce cyclooxygenases in chicken embryo fibroblasts.. Proc Natl Acad Sci U S A.

[PDF_00536] Mangiarotti R., Danova M., Alberici R., Pellicciari C. (1998). All-trans retinoic acid (ATRA)-induced apoptosis is preceded by G1 arrest in human MCF-7 breast cancer cells.. Br J Cancer.

[PDF_00539] Martin S. J., Finucane D. M., Amarante-Mendes G. P., O'Brien G. A., Green D. R. (1996). Phosphatidylserine externalization during CD95-induced apoptosis of cells and cytoplasts requires ICE/CED-3 protease activity.. J Biol Chem.

[PDF_00543] Mosmann T. (1983). Rapid colorimetric assay for cellular growth and survival: application to proliferation and cytotoxicity assays.. J Immunol Methods.

[PDF_00546] Oberhammer F., Wilson J. W., Dive C., Morris I. D., Hickman J. A., Wakeling A. E., Walker P. R., Sikorska M. (1993). Apoptotic death in epithelial cells: cleavage of DNA to 300 and/or 50 kb fragments prior to or in the absence of internucleosomal fragmentation.. EMBO J.

[PDF_00550] Pasricha P. J., Bedi A., O'Connor K., Rashid A., Akhtar A. J., Zahurak M. L., Piantadosi S., Hamilton S. R., Giardiello F. M. (1995). The effects of sulindac on colorectal proliferation and apoptosis in familial adenomatous polyposis.. Gastroenterology.

[PDF_00558] Piazza G. A., Rahm A. K., Finn T. S., Fryer B. H., Li H., Stoumen A. L., Pamukcu R., Ahnen D. J. (1997). Apoptosis primarily accounts for the growth-inhibitory properties of sulindac metabolites and involves a mechanism that is independent of cyclooxygenase inhibition, cell cycle arrest, and p53 induction.. Cancer Res.

[PDF_00554] Piazza G. A., Rahm A. L., Krutzsch M., Sperl G., Paranka N. S., Gross P. H., Brendel K., Burt R. W., Alberts D. S., Pamukcu R. (1995). Antineoplastic drugs sulindac sulfide and sulfone inhibit cell growth by inducing apoptosis.. Cancer Res.

[PDF_00567] Qiao L., Hanif R., Sphicas E., Shiff S. J., Rigas B. (1998). Effect of aspirin on induction of apoptosis in HT-29 human colon adenocarcinoma cells.. Biochem Pharmacol.

[PDF_00570] Ricchi P., Pignata S., Di Popolo A., Memoli A., Apicella A., Zarrilli R., Acquaviva A. M. (1997). Effect of aspirin on cell proliferation and differentiation of colon adenocarcinoma Caco-2 cells.. Int J Cancer.

[PDF_00573] Schwenger P., Alpert D., Skolnik E. Y., Vilcek J. (1998). Activation of p38 mitogen-activated protein kinase by sodium salicylate leads to inhibition of tumor necrosis factor-induced IkappaB alpha phosphorylation and degradation.. Mol Cell Biol.

[PDF_00577] Shao R. G., Shimizu T., Pommier Y. (1997). 7-Hydroxystaurosporine (UCN-01) induces apoptosis in human colon carcinoma and leukemia cells independently of p53.. Exp Cell Res.

[PDF_00586] Shiff S. J., Koutsos M. I., Qiao L., Rigas B. (1996). Nonsteroidal antiinflammatory drugs inhibit the proliferation of colon adenocarcinoma cells: effects on cell cycle and apoptosis.. Exp Cell Res.

[PDF_00583] Shiff S. J., Qiao L., Tsai L. L., Rigas B. (1995). Sulindac sulfide, an aspirin-like compound, inhibits proliferation, causes cell cycle quiescence, and induces apoptosis in HT-29 colon adenocarcinoma cells.. J Clin Invest.

[PDF_00580] Shiff S. J., Rigas B. (1997). Nonsteroidal anti-inflammatory drugs and colorectal cancer: evolving concepts of their chemopreventive actions.. Gastroenterology.

[PDF_00589] Smalley W. E., DuBois R. N. (1997). Colorectal cancer and nonsteroidal anti-inflammatory drugs.. Adv Pharmacol.

[PDF_00591] Smith W. L. (1989). The eicosanoids and their biochemical mechanisms of action.. Biochem J.

[PDF_00593] Subbegowda R., Frommel T. O. (1998). Aspirin toxicity for human colonic tumor cells results from necrosis and is accompanied by cell cycle arrest.. Cancer Res.

[PDF_00596] Tsujii M., DuBois R. N. (1995). Alterations in cellular adhesion and apoptosis in epithelial cells overexpressing prostaglandin endoperoxide synthase 2.. Cell.

[PDF_00599] Tsujii M., Kawano S., Tsuji S., Sawaoka H., Hori M., DuBois R. N. (1998). Cyclooxygenase regulates angiogenesis induced by colon cancer cells.. Cell.

[PDF_00605] Van Antwerp D. J., Martin S. J., Kafri T., Green D. R., Verma I. M. (1996). Suppression of TNF-alpha-induced apoptosis by NF-kappaB.. Science.

[PDF_00602] Vanags D. M., Pörn-Ares M. I., Coppola S., Burgess D. H., Orrenius S. (1996). Protease involvement in fodrin cleavage and phosphatidylserine exposure in apoptosis.. J Biol Chem.

[PDF_00610] Vane J. R. (1971). Inhibition of prostaglandin synthesis as a mechanism of action for aspirin-like drugs.. Nat New Biol.

[PDF_00612] Wang C. Y., Mayo M. W., Baldwin A. S. (1996). TNF- and cancer therapy-induced apoptosis: potentiation by inhibition of NF-kappaB.. Science.

[PDF_00615] Woo M., Hakem R., Soengas M. S., Duncan G. S., Shahinian A., Kägi D., Hakem A., McCurrach M., Khoo W., Kaufman S. A. (1998). Essential contribution of caspase 3/CPP32 to apoptosis and its associated nuclear changes.. Genes Dev.

[PDF_00619] Yin M. J., Yamamoto Y., Gaynor R. B. (1998). The anti-inflammatory agents aspirin and salicylate inhibit the activity of I(kappa)B kinase-beta.. Nature.

[PDF_00621] Zapata J. M., Krajewska M., Krajewski S., Huang R. P., Takayama S., Wang H. G., Adamson E., Reed J. C. (1998). Expression of multiple apoptosis-regulatory genes in human breast cancer cell lines and primary tumors.. Breast Cancer Res Treat.

[PDF_00607] van Engeland M., Nieland L. J., Ramaekers F. C., Schutte B., Reutelingsperger C. P. (1998). Annexin V-affinity assay: a review on an apoptosis detection system based on phosphatidylserine exposure.. Cytometry.

